# Influence of ursodeoxycholic acid on blood glucose, insulin and GLP-1 in rats with liver fibrosis induced by bile duct ligation

**DOI:** 10.1186/s13098-023-00989-z

**Published:** 2023-02-14

**Authors:** Xiu-Ping Bai, Wen-Jin Du, Hua-Bing Xing, Guo-Hua Yang, Rui Bai

**Affiliations:** 1 Endocrinology Division, The Second Hospital of Shanxi Medical University, Taiyuan, 030001 Shanxi China; 2 Central Laboratory, The Second Hospital of Shanxi Medical University, Taiyuan, 030001 Shanxi China

**Keywords:** Liver fibrosis, Glucose levels, Ursodeoxycholic acid, Genes

## Abstract

**Background:**

The prevalence of impaired glucose tolerance and diabetes is much higher in people with cirrhosis than that in the general population. However, there are inadequate concrete guidelines for the management of diabetes in these patients, particularly in the early stage. Bile aids (BAs) have been found to exert hormone-like functions in the control of lipid and glucose metabolism. We studied the effect of ursodeoxycholic acid (UDCA) on glucose levels in rats with cirrhosis induced by bile duct ligation (BDL).

**Methods:**

SD rats were divided into three groups: sham operation (Group A); BDL (Group B), and UDCA plus BDL (Group C). After 4 weeks, oral glucose tolerance tests were performed. Serum biochemical parameters and the levels of glucose, insulin, and glucagon-like peptide 1 (GLP-1) were measured. Histopathology of the liver and islet was observed. The gene expression of cholesterol 7α-hydroylase (CYP7A1), microsomal oxysterol 7a-hydroxylase (CYP7B1) in the liver, and Takeda G-protein-coupled receptor-5 (TGR5) in the intestine was determined by real-time PCR.

**Results:**

Compared with Group A, fasting glucose and 1-h and 2-h postprandial glucose levels increased slightly (all *P* > 0.05), 2-h postprandial insulin levels increased significantly (*P* < 0.05), 15 min postprandial GLP-1 levels decreased (*P* < 0.05) in Group B. Compared with Group B, fasting glucose and 1-h postprandial glucose levels decreased (all *P* < 0.05), 2-h postprandial insulin levels decreased (*P* < 0.01), and 15 min postprandial GLP-1 levels increased (*P* < 0.05) in Group C. After UDCA intervention, liver fibrosis induced by BDL was alleviated, and the islet areas were increased (*P* < 0.05). Compared with Group A, the mRNA expression of CYP7A1 and CYP7B1 in the liver increased, and the mRNA expression of TGR5 in the intestine decreased in Group B (all *P* < 0.05). Compared with Group B, the mRNA expression of CYP7A1 and CYP7B1 in the liver decreased, and TGR5 in the intestine increased in Group C (*P* < 0.05).

**Conclusions:**

After 4 weeks of BDL, the rats developed liver fibrosis and abnormal glucose metabolism. UDCA administration improved liver fibrosis, increased islet area, decreased glucose levels, inhibited genes in BA synthesis, enhanced TGR5 gene expression in the intestine, and further improved islet function.

## Background

The prevalence of diabetes mellitus in cirrhotic patients is much higher than that in the general population [1]. An association between diabetes mellitus (DM) and liver cirrhosis is well known, but the mechanism is not very clear. Additionally, treating diabetes is difficult in cirrhotic patients because of the metabolic impairments caused by liver disease and because the most appropriate pharmacologic treatment has not been defined [2].

Liver cirrhosis is the final pathological result of various chronic liver diseases, and fibrosis is the precursor of cirrhosis [3]. The management of liver cirrhosis is centered on the treatment of the causes and complications, including better screening for early fibrosis. Current evidence suggests that the process of hepatic fibrosis is driven primarily by the development of inflammation in response to parenchymal injury [4]. Important mechanisms for the reversibility of liver fibrosis are the cessation of chronic damage allowing hepatocyte recovery and modulating the microenvironment [5]. The histological evaluation of a liver biopsy is still the gold standard in assessing the stage of liver fibrosis [3], so it is not convenient to explore the mechanism of diabetes in people with cirrhosis, and we need to construct an animal model to explore it. The two most commonly used models of experimental fibrosis are toxic damage elicited by carbon tetrachloride (CCl4) and bile duct ligation (BDL) [6]. CCl4 is harmful to researchers, so we constructed an animal model of liver fibrosis induced by BDL.

Ursodeoxycholic acid (UDCA) is a hydrophilic bile acid that acts as a bile secretagogue and cytoprotective agent [7]. Administration of UDCA improves liver biochemistry and may delay histologic progression [8]. In recent years, in addition to helping intestinal absorption of dietary fats, fat-soluble vitamins, and other nutrients, bile acids (BAs) are also being recognized as signaling molecules in the human body because they are able to regulate metabolic and cellular functions by interacting with BA receptors [9].

We previously found that [10] after 4 weeks of BDL, the mice developed cirrhosis, while postprandial glucose levels increased after 2 h, and the mice developed insulin resistance. We want to know whether UDCA, in addition to improving liver histology, is also beneficial for improving glucose levels. Therefore, we constructed an animal model of liver fibrosis induced by BDL, observed the influence of UDCA intervention on glucose levels, and explored its possible mechanisms. We hope our study is helpful in finding an adequate management of DM in persons with liver cirrhosis and aiding us in clinical practice.

## Methods

### Animal model

Specific pathogen free (SPF) Male SD rats (6–8 weeks of age; weighing 150–200 g) were purchased from the Experimental Animal Center of ShanXi Medical University (TaiYuan, ShanXi, China) and adapted to the environment for 1 week, before study. All rats were housed in SPF colony cages (4 rats in each cage) with a 12 h light/dark cycle in a temperature-controlled environment. The rats were randomly divided into three groups according to the random number table method: the sham operation group (Group A, n = 10), the BDL group (Group B, n = 10), and the UDCA plus BDL group (Group C, n = 10). All rats were fed a normal chow diet (66.5% carbohydrates, 10.2% fat, 23.3% protein), and the rats in Group C received 0.25% (w/w diets food) UDCA (Shanghai Sangon Biological Engineering Technology and Services Co. Ltd. Shanghai, China; dissolved in normal saline) daily by gavage for 4 weeks according to the methods described [11, 12] with some modifications. Rats in Groups A and B received an equal amount of normal saline. Body weight and food intake were measured every other day. The animals had free access to food and drinking water. All animal care and experimental procedures were performed in accordance with the guidelines of the Animal Care Committee of ShanXi Medical University of China. BDL was used to induce liver cirrhosis in the rats and each experiment was performed in our laboratory. The rats were anesthetized with 0.5% pentobarbital (45 mg·kg^−1^ body weight) by an intraperitoneal ihjection before the operation. Under sterile conditions, the abdominal cavities of the rats in Groups A, B and C were opened, and the common bile ducts were isolated but not ligated in Group A rats. After surgery, the rats were placed into a single cage, and wound care was applied. The bile ducts of the rats in Groups B and C were ligated at the midpoint as previously described [13, 14]. The rats in the three groups were euthanized under anesthesia at 4 weeks after BDL.

### Oral Glucose tolerance tests (OGTTs) and insulin release tests

At 4 weeks after BDL, an OGTT was performed by glucose gavage (5 g D-glucose/kg body wt) after an overnight fast. The rat tail was pierced by a needle, and a drop of venous blood was taken to measure blood glucose. Another 300 µl tail vein blood was collected and centrifuged at 2000 rpm for 15 min to obtain serum for insulin measurement. Blood glucose and insulin were monitored after glucose gavage for 0 h, 1 h, and 2 h, and blood glucose was monitored using a glucometer (Roche Diagnostics, Switzerland). Insulin concentrations were determined using a rat insulin enzyme-linked immunosorbent assay kit (RD system) using a rat insulin standard. β cell function was assessed by the homeostasis model (HOMA) as previously described [15]. HOMA-β was calculated according to the formula: 20 × fasting insulin (µIU/ml)/(fasting plasma glucose (mmol/L) − 3.5) [16].

### Biochemical analysis

At 4 weeks after BDL, under anesthesia, blood samples were collected from the abdominal aorta and centrifuged at 3000 rpm for 15 min to obtain serum. Serum concentrations of triglycerides (TG), total bile acids (TBA), alanine aminotransferase (ALT), and aspartate aminotransaminase (AST) were determined using a microplate (Reitman-Frankel colorimetric assay) according to the manufacturer’s protocol (Nanjing Jiancheng Corp, NanJing, China).

### Serum glucagon-like peptide 1 (GLP-1) measurement

Rats were made to fast 4–5 h prior to the experiment and received an oral gavage with sitagliptin (160 mg/kg) according to the reference [17]. Blood was collected after glucose gavage (5 g D-glucose/kg body wt) for 0 min, 15 min, 30 min, and 60 min and centrifuged at 3000 rpm for 15 min to obtain serum. GLP-1 concentrations were determined using a rat GLP-1 enzyme-linked immunosorbent assay kit (detection limit: 0.15 pmol/l-4.00 pmol/l, the coefficient of variation (CV) of inter-assay: < 10%, and CV of intra-assay: < 12%) using a rat GLP-1 standard according to the manufacturer’s instruction (Jiangsu Meimian Industrial Co., Ltd. Jiangsu, China). Serum samples were diluted, the concentrations obtained from the standard curve were multiplied by the dilution factor when analyzing the results.

### Histopathology of the liver and islet

At the end of the experiment, the rats in the three groups were euthanized under anesthesia. Livers and pancreases (n = 5) were selected from the three groups randomly according to the method of random number table, rapidly rinsed with PBS and immersed in 10% formaldehyde (Shanghai Sangon Biological Engineering Technology and Services Co. Ltd. Shanghai, China). Specimens were fixed in 10% formaldehyde, and then embedded in paraffin, serially sectioned (4 μm) and stained with hematoxylin and eosin (HE) to assess liver and islet morphology, and Masson's trichrome to assess liver collagen content, respectively. Images were analyzed using Image‐Pro Plus software (Media Cybernetics, Rockville, MD, USA) [18]. The areas of the islets were measured per visual field under a microscope at 20-fold magnification.

### Quantitative Real-time PCR analysis

Total RNA was extracted, and reverse transcription was performed with 460 ng of total RNA in a total volume of 10 µl under conditions of 37 °C for 15 min, 85 °C for 5 s and 4 °C for 5 min. cDNA was synthesized using the Reverse Transcriptase kit according to the manufacturer’s protocol (PrimeScript RT Master Mix, RR036A, Takara, Biomedical Technology Co., Ltd.), and the cDNA product was then amplified by real-time PCR in a total volume of 20 µl according to the manufacturer’s protocol (SYBR premix Ex Taq™ II, RR820A, Takara, Biomedical Technology Co., Ltd) using gene-specific primers (Table 1) on an ABI 7300/7500 real-time PCR instrument (Applied Biosystems, Carlsbad, CA, USA). To 2 μl of cDNA, 0.8 μl of primers for the gene of interest and 0.8 μl of primers for the reference gene were added, and the reaction conditions were 40 cycles of 95 °C for 30 s, 95 °C for 5 s, and 55 °C–60 °C for 34 s. Relative mRNA expression levels were calculated using the ΔΔCq method and normalized to β-actin mRNA levels. Individual samples were assayed in triplicate, and the average quantification cycle (Cq) was calculated for the gene of interest and the reference gene. Based on the difference between both Cq values, the comparison was calculated. All primers were synthesized by Shanghai Sangon Biological Engineering Technology and Services Co. Ltd. (Shanghai, China). The primer efficiencies were 99.98%-101.01%.

**Table 1:** Primer used for qPCR

Gene	Accession number	Nucleotide sequence (from 5′ to 3′)	Amplicon (bp)
*CYP-7A1*	NM-012942.2	F:TGGCTGTCATGTGCAGGCGCCAA R:ACGCACTGCCTCAGCAAACACACA	159
*CYP7B1*	NM_019138.1	F: ACCACAGTCGCATGTTTCTGGGCA R: TCCGCTAAGCTTCTCTGCCACCCT	108
*TGR5*	NM_177936.1	F:GCCCGCTGTGGGGGCCACTGCCCT R:GGGTGCATCACGGCACACCGCCCGC	109
*β-actin*	XM-032887061.1	F:AAGTCCCTCACCCTCCCAAAAG R: AAGCAATGCTGTCACCTTCCC	96

*CYP7A1* Cholesterol 7α-hydroylase, *CYP7B1* Microsomal oxysterol 7a-hydroxylase, *TGR5* Takeda G-protein-coupled receptor-5

### Statistical analyses

Values for normal distributions are presented as mean ± standard deviation (SD), and for non-normal distributions are transferred by logarithm to accord with normal distributions and are presented as median (range). Statistical analysis was performed with the Statistics Package for Social Science 19 (SPSS 19). The average difference in parameters among the three groups was analyzed by an ANOVA. Differences were considered significant at *P* < 0.05.

## Results

### Physical conditions and serum biochemical analysis

After BDL treatment, the rats in Group B gradually developed jaundice and yellowish pigmentation of the sclera, the color of the urine became yellow, and the color of the stool became white. The rats in Group A were in good condition. The rats in Group C were improved compared with those in Group B.

After 4 weeks, the levels of serum ALT, AST, TBA and TG were not significantly different among the three groups (*P* > 0.05) (Table 2).

**Table 2:** Serum biochemical analysis after BDL for 4 weeks

Index	Group A (n = 10)	Group B (n = 10)	Group C (n = 10)
ALT(U/L) (Q1–Q3)	35.85(17.10–61.90)	37.56(18–79.92)	39.67(14.84–68.98)
AST (U/L) (Q1–Q3)	43.99(32.63–62.30)	39.67(24.48–55.03)	48.56(37.54–64.09)
TBA(umol/l) (Q1– Q3)	72.17(71.17–164.42	96.90(80.71–129.59)	95.90(85.36–116.77)
TG (mmol/l)	1.61 ± 1.10	1.35 ± 1.21	0.99 ± 0.52

*TG* triglycerides; *ALT* alanine aminotransferase; *AST* aspartate aminotransaminase; *TBA* total bile acids. *Group A* Sham operation group; *Group B* BDL group; *Group* *C* UDCA plus BDL group

### OGTT and insulin release test

After 4 weeks of BDL, compared with Group A, Group B’s fasting glucose, 1 h and 2 h postprandial glucose increased to some extent (fasting: 5.25 ± 1.00 vs. 4.63 ± 0.87 mmol/l; 1 h: 7.10 ± 0.96 vs. 7.05 ± 0.10 mmol/l; 2 h: 7.19 ± 1.21 vs. 6.65 ± 0.38 mmol/l, all *P* > 0.05). Compared with Group B, Group C’s fasting and 1 h postprandial glucose decreased significantly (fasting: 4.10 ± 1.11 vs. 5.25 ± 1.00 mmol/l*;* 1 h: 5.59 ± 1.43 vs. 7.10 ± 0.96 mmol/l, all *P* < 0.05), and 2 h postprandial glucose decreased slightly (6.36 ± 2.25 vs. 7.19 ± 1.21 mmol/l, *P* > 0.05). Compared with Group A, fasting and 1 h postprandial insulin levels were not obviously changed in Group B (fasting: 3.75 ± 1.24 vs. 4.75 ± 0.42; 1 h: 4.48 ± 1.56 vs. 4.57 ± 0.94 miu/l, all *P* > 0.05), but 2 h postprandial insulin levels increased significantly (7.15 ± 3.52 vs. 4.33 ± 1.36 miu/l, *P* < 0.05).

**Fig.1:**
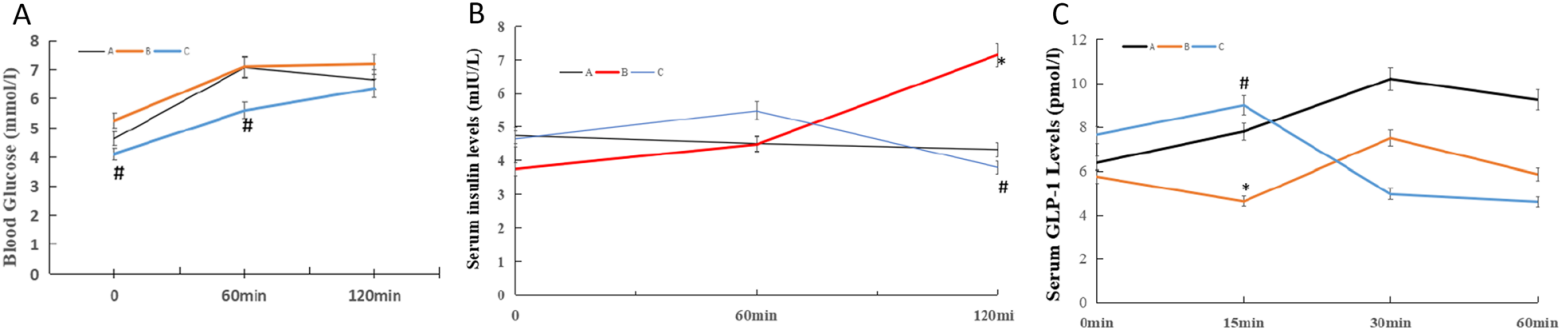
OGTTs, insulin release tests and GLP-1 levels. **A** OGTTs after 4 weeks of BDL; **B** Insulin release tests after 4 weeks of BDL; **C** Serum GLP-1 levels during OGTT. black line, Group **A** (sham operation group); red line, Group **B** (BDL group); blue line, Group **C** (UDCA plus BDL group)

Compared with Group B, fasting and 1 h postprandial insulin levels were not obviously changed in Group C (fasting: 4.65 ± 1.13 vs. 3.75 ± 1.24; 1 h: 5.48 ± 2.18 vs. 4.48 ± 1.56 miu/l, all *P* > 0.05), but 2 h postprandial insulin levels decreased significantly (3.80 ± 2.09 vs. 7.15 ± 3.52 miu/l, *P* < 0.01) (Fig. 1).

**Fig. 2:**
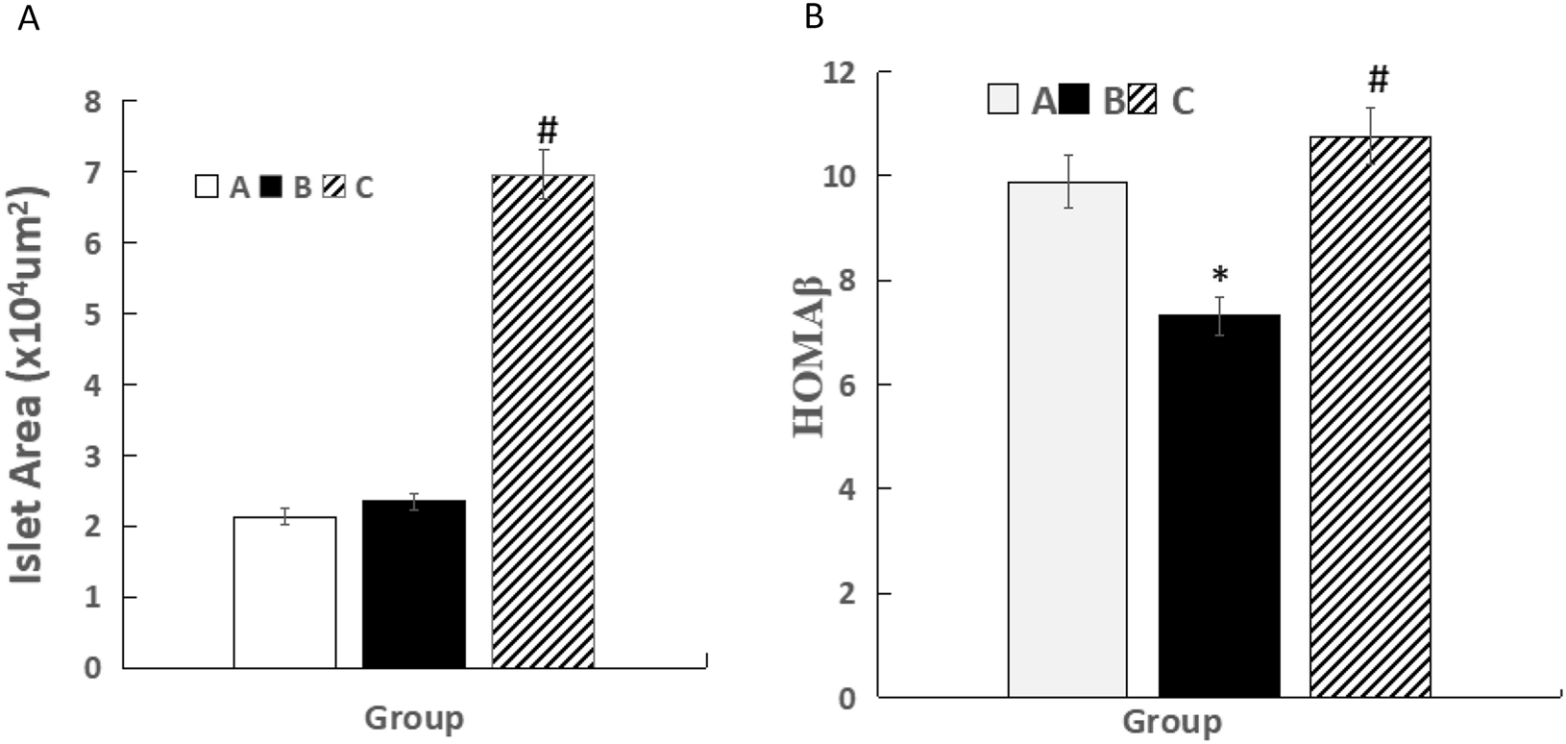
Comparison of the islet areas and HOMA β in the three groups (n = 5).* *P* < 0.05 vs Group **A** (sham operation group), ^#^*P* < 0.05 vs Group **B** (BDL group). white squares, Group **A** (sham operation group); black square, Group **B** (BDL group); black diagonal width on white background, Group C (UDCA plus BDL group)

Compared with Group A, HOMA β was decreased in Group B (7.32 ± 2.73 vs. 9.89 ± 1.07 miu/mmol, *P* < 0.05). Compared with Group B, HOMA β was increased in Group C (10.76 ± 5.81 vs. 7.32 ± 2.73miu/mmol, *P* < 0.05) (Fig. 2).

### Serum GLP-1 levels in the three groups

After 4 weeks of BDL, compared with Group A, Group B’s baseline GLP-1 levels changed slightly (6.38 ± 1.45 vs. 5.75 ± 2.55 pmol/l, *P* > 0.05), 15 min postprandial GLP-1 levels decreased significantly (7.80 ± 1.75 vs. 4.62 ± 1.25 pmol/l, *P* < 0.05), and 30 min and 60 min postprandial GLP-1 levels changed slightly (30 min:10.20 ± 0.50 vs. 7.50 ± 2.40 pmol/l; 60 min: 9.25 ± 0.30 vs. 5.85 ± 2.55 pmol/l, all *P* > 0.05). Compared with Group B, Group C’s baseline GLP-1 levels increased to some extent (7.65 ± 1.90 vs. 5.75 ± 2.55 pmol/l, *P* > 0.05), 15 min postprandial GLP-1 levels increased significantly (9.00 ± 1.30 vs. 4.62 ± 1.25 pmol/l, *P* < 0.05), and 30 min and 60 min postprandial GLP-1 levels changed slightly (30 min: 4.95 ± 2.25 vs. 7.50 ± 2.40 pmol/l; 60 min: 4.60 ± 2.05 vs. 5.85 ± 2.55 pmol/l, all *P* > 0.05) (Fig. 1).

### Morphological changes in the liver and islet

**Fig.3:**
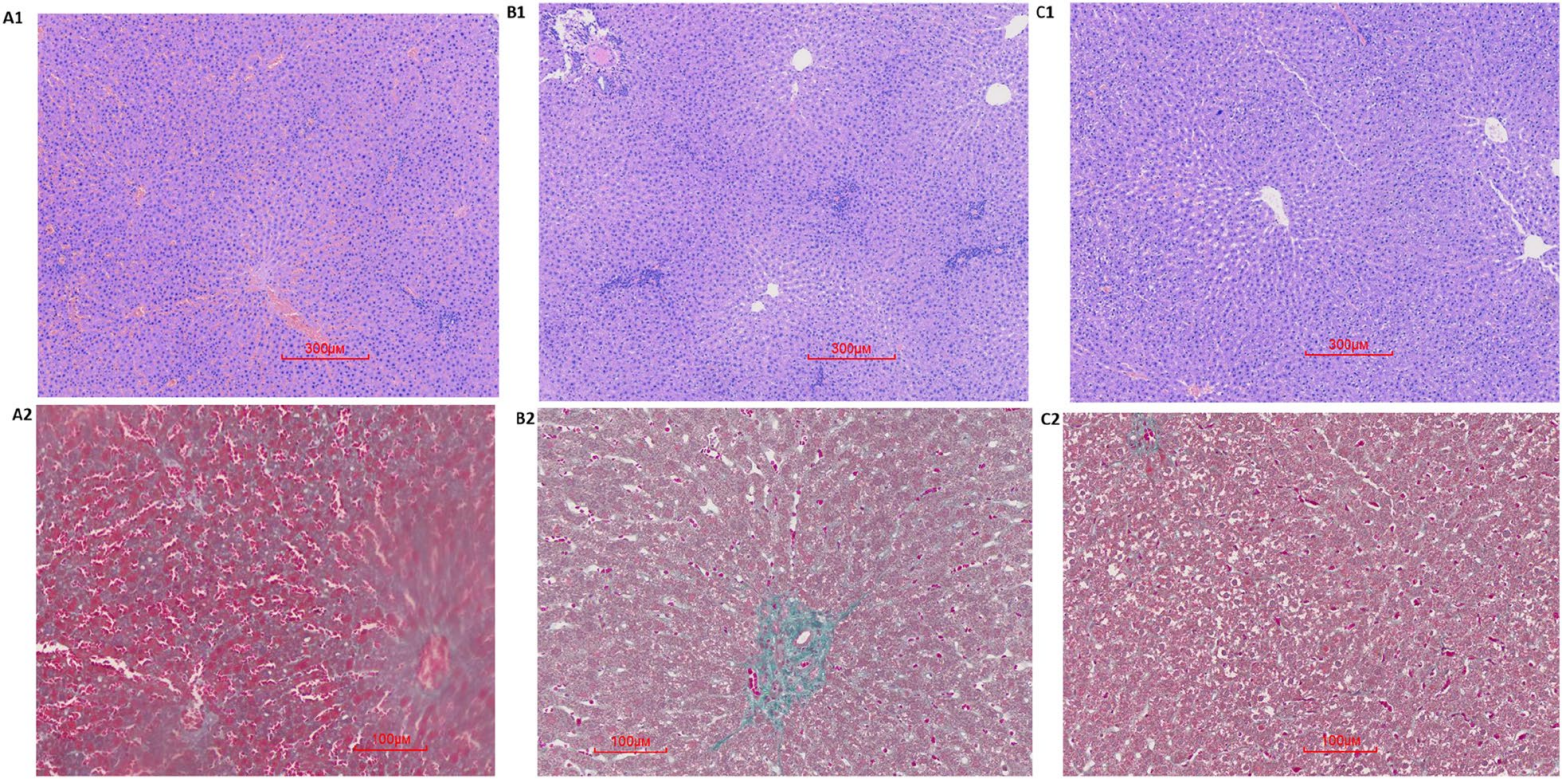
Histological assessment of the liver using hematoxylin and eosin and Masson’s trichrome staining. Liver tissues were stained with HE in the three groups at week 4 (**A1**, **B1**, **C1**) **A1** sham operation group at week 4 (HE 40×); **B1** BDL group at week 4 (HE 40×); **C1**, UDCA plus BDL group at week 4 (HE 40×). liver tissues were stained using MTC in the three groups at weeks 4 (**A2**, **B2**, **C2**). **A2** sham operation group at week 4 (MTC 100×); **B2** BDL group at week 4 (MTC 100 ×); **C3** UDCA plus BDL group at week 4 (MTC 100×). Scale bars: 300 μm, HE: Hematoxylin and eosin; Scale bars: 100 μm, MTC: Masson’s trichrome. (Images were randomly chosen from 5 rats in the three groups, n = 5) (The data are representative of 5 rats in the three groups, n = 5)

**Fig. 4:**
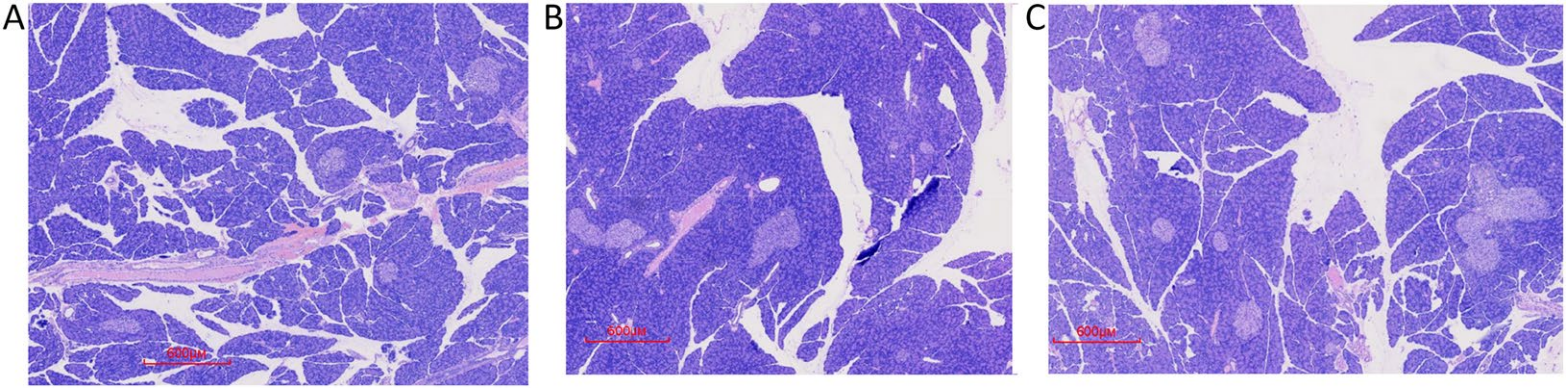
Histological assessment of islets using hematoxylin and eosin. Pancreatic tissues were stained with HE in the three groups at weeks 4. (**A**–**C**). **A** sham operation group at week 4 (HE 20×); **B** BDL group at week 4 (HE 20×); **C** UDCA plus BDL group at week 4 (HE 20×). Scale bars: 600 μm; (Images were randomly chosen from 5 rats in the three groups, n = 5; the data are representative of 5 rats in the three groups, n = 5)

After 4 weeks, as indicated by HE staining, BDL induced liver fibrosis, bile duct extension, disordered hepatic lobule structure in Group B, compared with that of the group A. BDL-induced hepatic fibrosis was further evaluated through Masson staining, which is a classical histopathological technique used for observing collagen. In Group B rats, extensive accumulation of collagen (blue color staining) was observed, and compared with Group B, liver fibrosis, bile duct extension, and hepatic lobule structural disorder were obviously improved in Group C (Fig. 3). Compared with Group A, the areas of the islets were changed slighty [(2.13 ± 1.99 vs 2.34 ± 1.07) × 10^4^ µm^2^, *P* > 0.05] in Group B, and compared with Group B, the areas of the islets were increased [6.96 ± 2.43 vs 2.34 ± 1.07) × 10^4^ µm^2^, *P* < 0.05] in Group C (Figs. 2, 4).

### Gene expression of cholesterol 7α-hydroylase (CYP7A1), microsomal oxysterol 7a-hydroxylase (CYP7B1) in the liver, Takeda G-protein-coupled receptor-5 (TGR5) in the intestine

**Fig. 5:**
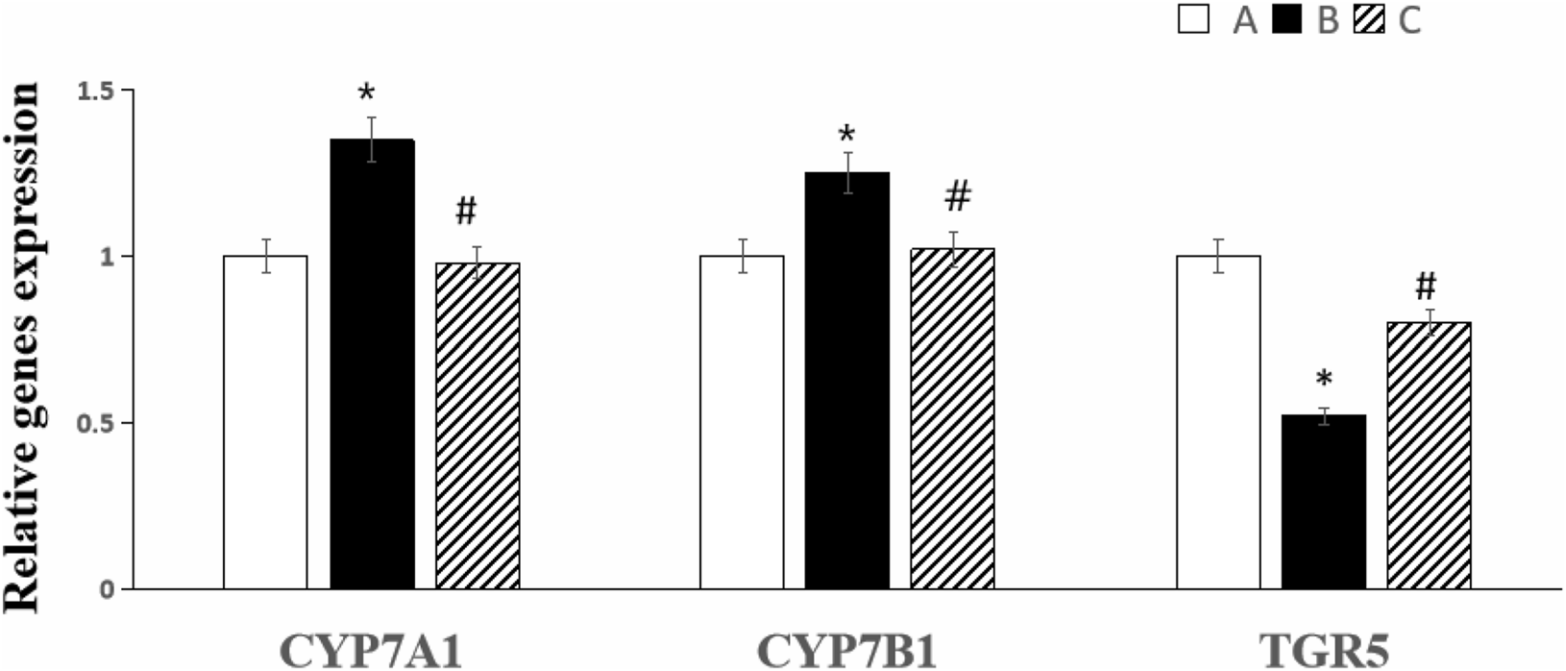
Comparison of CYP7A1 and CYP7B1 mRNA expression in the liver, TGR5 mRNA expression in the intestine in the three groups (n = 3). *P < 0.05 vs Group **A** (sham operation group), ^#^*P* < 0.05 vs Group **B** (BDL group). white squares, Group **A** (sham operation group); black square, Group **B** (BDL group); black diagonal width on white background, Group **C** (UDCA plus BDL group)

Compared with Group A, the mRNA expression of CYP7A1 and CYP7B1 in the liver in Group B increased by approximately 35% and 25%, respectively (all *P* < 0.05). Compared with Group B, the mRNA expression of CYP7A1 and CYP7B1 in the liver in Group C decreased by approximately 37% and 23%, respectively (all *P* < 0.05). Compared with Group A, the mRNA expression of TGR5 in the intestine in Group B decreased by approximately 48%. Compared with Group B, it increased by approximately 28% in Group C (*P* < 0.05) (Fig. 5).

## Discussion

In addition to aiding the absorption of lipid-soluble nutrients from the intestine, BAs exert hormone-like functions in the control of lipid and glucose metabolism. BAs are synthesized in the liver from cholesterol in classic and alternative pathways. The classic pathway is initiated by cholesterol 7α-hydroxylase (CYP7A1), the only rate-limiting enzyme in bile acid synthesis, and synthesizes two primary bile acids, cholic acid (CA) and chenodeoxycholic acid (CDCA), in the liver. Most bile acid synthesis occurs in the classical pathway. The alternative pathway is initiated by sterol 27-hydroxylase (CYP27A1), followed by oxysterol 7α-hydroxylase (CYP7B1) to produce mainly CDCA [19]. The 7α-hydroxyl groups in CDCA can also be epimerized to 7β to form ursodeoxycholic acid (UDCA) [20]. Primary BAs are performed by gut bacteria and finally result in the formation of secondary BAs such as deoxycholic acid (DCA) and lithocholic acid (LCA) and tertiary BAs such as UDCA. In the gastrointestinal tract, BAs, by activating membrane G protein-coupled bile acid receptor (TGR5), stimulate GLP-1 secretion from enteroendocrine L-cells, which stimulates insulin secretion from pancreatic β-cells [21].

UDCA has been used in the treatment of liver disease [22], with beneficial effects in alleviating liver damage. UDCA has been recognized as a ligand of TGR5, and TGR5-mediated bile acid sensing controls glucose homeostasis. Some researchers have reported that UDCA lowers glucose levels [23], but we have not observed the effect of UDCA on blood glucose related to cirrhosis, and this mechanism has not been elucidated.

Our study showed that after 4 weeks of BDL, the rats in Group B gradually developed jaundice, a decrease in appetite and low spirits. Serum biochemical analysis indicated that the levels of AST, ALT and TBA were not changed significantly. These biochemical changes are similar to previous findings in humans [24], but these results are different from Shakerinasab et al. and Moslemi et al.’s reports [25, 26] that SD rats after BDL for 7 days, AST, ALT and TBA were increased, also Nasehi Z, et al. [27] found the levels of serum AST, ALT and TBA were increased in SD rats after BDL for three weeks. Ghallab A, et al.[28] found that ALT, AST and TBA increased in the acute phase, reaching a peak on days 1 and 2 after BDL, but decreased significantly below control levels in the chronic phase, with normalization on day 21. The differences of our findings from those previous reports may be owed to the different time points of detection after BDL, we measured these indexes in the chronic phase, but they measured in the acute phase.

Liver pathology showed accumulation of fibrin, extension of the bile duct, and a slightly disordered structure of hepatic lobules in rat liver tissues. These characteristics suggested that these rats developed liver fibrosis, the early stage of cirrhosis, by BDL, and these findings are consistent with previous reports [28]. Compared with Group B, liver fibrosis obviously improved in Group C.

The measurement of blood glucose showed that, compared with Group A, Group B’s fasting glucose, 1 h and 2 h postprandial glucose levels increased slightly, and 2 h postprandial insulin levels increased significantly, suggesting that these rats developed postprandial hyperinsulinemia, and these changes are similar to previous reports [29]. After UDCA administration, Group C’s fasting glucose and 1 h postprandial glucose levels decreased significantly, 2 h postprandial glucose levels decreased slightly, and 2 h postprandial insulin levels decreased significantly, suggesting that UDCA administration decreased glucose levels and alleviated hyperinsulinemia.

We then measured GLP-1 levels in the three groups and found that, compared with Group A, Group B’s 15 min postprandial GLP-1 levels decreased significantly, and compared with Group B, Group C’s levels increased significantly. This suggests that in the rats in the early stage of cirrhosis, the early phase of serum GLP-1 concentrations decreased and UDCA administration increased it.

We then detected the mRNA expression of TGR5 in the intestine in the three groups.

We found that TGR5 mRNA expression was decreased in Group B compared with Group A, and UDCA administration increased TGR5 mRNA expression in Group C. Our findings are consistent with Thomas C et al.’s [30] reports that UDCA acted as an agonist of TGR5 and increased GLP-1 secretion. We next measured the mRNA expression of CYP7A1 and CYP7B1 in the liver. We found that compared with Group A, CYP7A1 and CYP7B1 mRNA expression increased in Group B, suggesting that BDL increased the expression of genes responsible for BA synthesis in both the classic and alternative pathways. Our findings are consistent with previous reports [31]. After UDCA administration, the mRNA expression of CYP7A1 and CYP7B1 decreased. This result suggested that UDCA administration decreased the expression of genes related to bile acid synthesis in the liver, this may further lead to the decrease of BA overload, which is harmful to liver cells [32].

Accumulation of high amounts of toxic bile acids could cause liver inflammation and injury and cholestatic liver diseases [33]. UDCA administration may be related to liver histological improvement, and our findings are similar to those of a previous report [34].

## Conclusion

We found that, after BDL, the rats developed liver fibrosis, decreased islet areas and postprandial hyperinsulinemia, and glucose levels had a trend to increase. UDCA administration decreased glucose levels, increased serum GLP-1 levels, alleviated hyperinsulinemia, increased islet areas, and further improved islet function in liver fibrosis rats. These changes may be related to its roles in enhancing TGR5 gene expression in the intestine, inhibiting the expression of genes in BA synthesis, and improving liver fibrosis. This suggests that UDCA may be used in the treatment of hyperglycemia in cirrhotic patients, and it is necessary to test this hypothesis in clinical research in the future. Our findings are important in providing evidence for experimental studies in the treatment of hyperglycemia caused by chronic liver disease.

## Data Availability

The datasets used and/or analysed during the current study are available from the corresponding author on reasonable request.

## Abbreviations

DM: Diabetes mellitus
BDL: Bile duct ligation
UDCA: Ursodeoxycholic acid
BAs: Bile acids
SPF: Specific pathogen free
OGTTs: Oral Glucose tolerance tests
HOMA: Homeostasis model
HOMA-β: HOMA of β cell function
TG: Triglycerides
TBA: Total bile acids
ALT: Alanine aminotransferase
AST: Aspartate aminotransferase
TG: Triglycerides
GLP-1: Glucagon-like peptide 1
HE: Hematoxylin and eosin
CYP7A1: Cholesterol 7α-hydroylase
CYP7B1: Microsomal oxysterol 7a-hydroxylase
TGR5: G-protein-coupled receptor-5
HGP: Hepatic glucose production
CA: Cholic acid
CDCA: Chenodeoxycholic
CYP27A1: Sterol 27-hydroxylase
DCA: Deoxycholic acid
LCA: Lithocholic acid
CV: Coefficient of variation
